# Revealing a safer sex option to reduce HIV risk: a cluster-randomized trial in Botswana

**DOI:** 10.1186/s12889-019-6844-8

**Published:** 2019-05-21

**Authors:** Noam Angrist, Mogomotsi Matshaba, Lesego Gabaitiri, Gabriel Anabwani

**Affiliations:** 1 Blavatnik School of Government, University of Oxford, 120 Walton St, Oxford, OX2 6GG United Kingdom; 2 Young 1ove, Gaborone, Botswana; 3 Baylor College of Medicine, Department of Pediatrics, Section of Retrovirology, Houston, TX USA; 4 Botswana-Baylor Children’s Clinical Centre of Excellence, Gaborone, Botswana; 5 Department of Statistics, University of Botswana, Gaborone, Botswana

**Keywords:** Impact evaluation, Botswana, HIV, Teen pregnancy, Health, School, Sex education, Southern Africa, Risk reduction

## Abstract

**Background:**

1.8 million new HIV infections occur every year, disproportionately affecting adolescent girls and young women. Abstinence-only risk avoidance approaches have had limited impact on reducing new infections. This cluster-randomized trial examines a risk reduction approach to curbing risky sex for school-going girls in Botswana.

**Methods:**

The unit of randomization was the school (*n* = 229). Intervention participants received a 1-h intervention revealing a safer sex option: dating same-age partners which have 5-9x lower HIV prevalence than older partners. Primary outcomes were pregnancy as a proxy for unprotected sex and HIV. Secondary outcomes included self-reported sexual behavior. Generalized linear multilevel models with school-level robust variance for adjusted relative risk ratios were used in an intention-to-treat analysis.

**Results:**

At a 12-month follow up, the intervention reduced pregnancy with an adjusted Relative Risk Ratio (aRRR) of .657 [95% CI .433–.997] significant at the 5% level. Effects were largest at junior school (aRRR = .575 [95% CI .394–.841]) and in rural areas (aRRR = .518 [95% CI .323–.831]), significant at the 1% level. There were no significant effects for primary school students, suggesting age of sexual debut and related mechanisms are critical factors in the intervention’s effectiveness. Moreover, baseline beliefs of which partner is riskiest mediate the magnitude of effects.

**Conclusions:**

Information on safe sex options can change sexual behavior. The success of the intervention working across contexts will depend on various factors, such as age of sexual debut and baseline beliefs.

**Trial registration:**

Pan African Clinical Trials Registry PACTR201901837047199. Registered 31 December 2018. Retrospectively registered. This study adheres to CONSORT guidelines.

## Background

17.8 million women aged 15 years or older were living with HIV in 2017. New infections are concentrated in sub-Saharan Africa at 64% [1]. Adolescent girls are disproportionately affected by the HIV epidemic, accounting for over three quarters of new infections in sub-Saharan Africa and contracting HIV 5–7 years earlier than their male counterparts [2, 3].

Recent advances in medical treatment have curbed death rates linked to HIV with 63% of those infected on anti-retroviral (ARV) treatment. However, new HIV infections persist with 1.8 million new infections in 2017 [2]. The majority of new infections occur through sexual intercourse [3]. In the face of millions of new infections and the significant financial costs of medication, sexual behavior change interventions are critical in combatting HIV/AIDS. Yet the evidence on sexual health education interventions that work is sparse.

The dominant approach to prevention for youth has focused on abstinence campaigns [4]. This approach is rooted in cultural, religious, and social factors. The abstinence message emphasizes extreme risk avoidance (“don’t have sex until marriage”). While optimal in theory, many young people do not abstain in practice [4]. In the absence of attractive safe sex options, they are left with one option: risky sex. Thus, abstinence-only messaging is missing a margin of impact by failing to provide a safe option to sexually active youth that would prefer a safe sex option if offered. A shift in higher numbers of youth to “low risk” safer sexual encounters could yield greater social and public health returns than minimal or no shifts to “no risk” scenarios. Such risk reduction approaches have met with success in other domains, including smoking and drugs injection. Examples include nicotine patches and supervised injection sites, which have significantly reduced smoking and mortality [5–7].

In the context of sexual behavior, risk reduction approaches have proved effective in high-income countries. A systematic review of quasi-experimental and randomized trial experiments showed that messaging covering safe sex options as compared to abstinence-only programming had positive impacts [8]. A review of 56 studies found that abstinence-only programs had no effect, while two-thirds of comprehensive programs increased delay in sexual debut and condom usage [4, 8].

However, limited rigorous evidence of risk reduction for sexual behavior exists in sub-Saharan Africa, where the burden of HIV is greatest. Much of the published research has relied on non-randomized evaluation methods, lacked statistical power, or used biased self-reported measures, providing only suggestive evidence [9–16]. A systematic review by the World Health Organization found limited evidence of sexuality education interventions showing casual impact on behavioral outcomes [17]. Despite limited rigorous evidence, the evidence that does exist suggests risk reduction approaches are likely to be effective in sub-Saharan Africa. A study across 22 sub-Saharan African countries found no association between PEPFAR-funded abstinence-only programming and reduced pregnancy or risky sexual behavior [18]. In contrast, comprehensive sexuality education has shown promise [4]. A notable example is a randomized trial in Kenya which revealed younger partners were a safer sex option than older partners, reducing pregnancy by 28%, a proxy for unprotected sex and HIV [19].

Our study builds on this literature with a cluster-randomized controlled trial of a risk-reduction intervention in Botswana. Botswana is a critical setting for curbing risky sex. The HIV prevalence rate is the third highest in the world, at 23.4% on average for adults [20]. Moreover, 60% of youth 19 or older are sexually active [21]. Since many adolescents are not abstaining, providing a safe sex alternative that has a high likelihood of take-up is relevant and has potential to be highly effective.

We study a specific risk reduction approach: encouraging dating of same-age partners instead of older partners. This is a potentially attractive and realistic option and dramatically safer. HIV rates in Botswana increase exponentially with age: 3.6% of 15–19 year-old boys are infected with HIV relative to 43.8% in 40–44 year old men [20]. Between ages 10 to 24, girls have a 3x increase in HIV prevalence, while HIV prevalence in their male peers remains constant. Older partners are associated with a series of additional risks in addition to HIV prevalence, such as unequal negotiation power, low condom usage and teen pregnancy [22, 23]. Yet, many young girls date older partners, unaware that the risk is an order of magnitude higher.

A study in Botswana found that 23.3% of young women in urban areas were having sex with older partners [22]. The pervasiveness of these relationships with older partners is driven by a few factors. In some instances, coercion or economic circumstances are the driving cause [22, 23]. However, evidence suggests many girls opt for older men, expressing agency over entering into the relationship [23]. These partners offer tangible, immediate benefits in the form of gifts, while the costs – such as the risk of unprotected sex, pregnancy or contracting HIV - are underestimated, long-term and not visible. The baseline in this study corroborates this lack of risk perception: 90% of youth could not identify that forty-year-old men, 45% of whom have HIV, were the highest risk group. In a series of focus groups, girls perceived these older men as low risk describing them as “mature and financially secure.”

In this context, revealing that older partners are 5-9x more likely to have HIV may elevate the cost of older partners as immediate and acute, as well as present a relatively safer sex option with lower-risk age-mates. Among age-mates, a variety of factors culminate in safer sex, including equalized negotiation power, both parties more likely to want safe sex, and HIV prevalence is significantly lower. This study aimed to evaluate the effectiveness of a 1-h module delivered in government schools in Botswana focused on this risk reduction approach.

The focus of the study was on adolescents. Preventing HIV infection among adolescents is critical for epidemic control. A significant segment of the population in Africa is young with nearly 60% of the population below the age of 25 [24]. Moreover, there has been near elimination of vertical HIV transmission from mother to child. For example, in Botswana the percentage of mother-to-child transmission rate was just 2% in 2013 [25]. To this end, there is a generation being born HIV-free. With effective prevention, the young generation can remain HIV-free and accelerate epidemic control.

## Methods

### Setting and recruitment

The study was implemented in Botswana across four regions in a third of the nation. The regions were: Kgatleng, Kweneng, South East and Southern. These regions were selected to be representative across urban and rural settings and across socioeconomic background. South East is the region where the capital, Gaborone, is located and is most urbanized within the country.

Primary and junior secondary schools were randomized to treatment or control from a list of schools provided by the Ministry of Education. In primary schools, all grade 6 students were reached or surveyed. In junior secondary schools, all grade 8 and 9 students were reached or surveyed. Primary school students progress from grade 6 to 7 in the same school while Junior secondary school students transition to a new senior secondary school after completing grade 10. This enabled tracking of students a year later. Intervention took place at the class-level at an hour convenient to the school. In junior schools, this often occurred during study hour. The ratio of instructor to student was on average 1:30 in primary schools and 1:40 in junior schools.

### Study population and retention

**Fig. 1:**
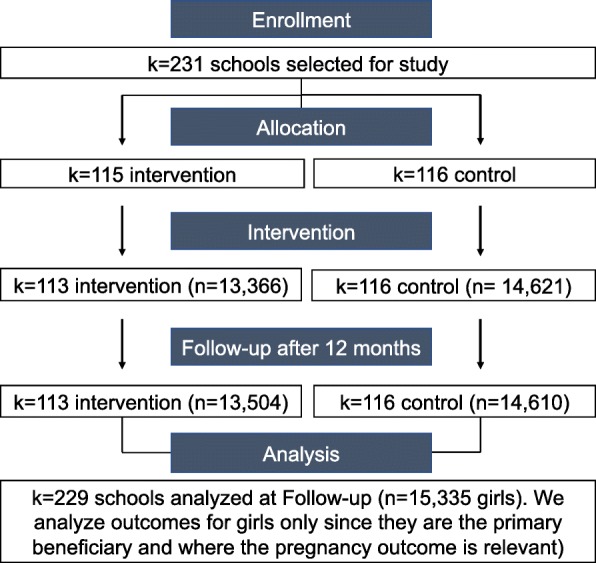
Participant Flow Chart

Baseline data and follow-up data was collected, respectively, from August to November, 2014 and from September–December, 2015 using standard pretested data collection forms. This enabled a 12-month follow-up. A random sample of 231 schools was selected from the Ministry of Education list within each region. Sample size was determined through power calculations in Stata 15 (STATACORP. College Station, TX). Two schools initially recorded as public schools were later verified as private schools and excluded since the study focused on government schools. The remaining sample of 229 schools were randomized to intervention or control. All schools and grades were successfully followed up. The baseline sample includes 229 schools (47 junior secondary, 182 primary). The follow-up sample includes all 229 schools (47 junior secondary, 182 primary). The final sample of analysis included only girls (229 schools, *n* = 15,335) as summarized in Fig. 1. We analyze outcomes for girls only since they are the primary beneficiary and where the pregnancy outcome is relevant. Some differences in the samples are due to idiosyncratic factors such as daily attendance variation.

### The intervention group

The intervention was a 1-h class revealing the relative HIV risks of older versus younger partners. At baseline, over 90% of students guessed this wrong as they thought older sex partners were safest. Thus, at the outset, the students did not realize dating same-age partners was a viable safer sex alternative. The intervention revealed this safer sex strategy through five segments: ice-breakers to generate comfort talking about sex; a puzzle activity where youth guessed in groups which age group of men was most likely to have HIV; a reveal of the true HIV graph disaggregated by age with statistics pulled from the Botswana AIDS Impact Survey (2013); a 14-min video produced by UNICEF showcasing a young girl overcoming an older partner in a rural setting; and finally, a short group discussion on the risks of intergenerational sex as well as actionable lessons learned to avoid risky older partners, including community support to draw on.

A related effect of the intervention is to change the perceived cost-benefit of dating older partners. At the outset, the benefits of older partners are known, visible and immediate, such as gifts in the form of airtime for cell phones. However, the costs, such as HIV, are underestimated and long-term. The intervention makes the HIV cost known and salient, thus potentially changing the cost-benefit of dating older partners.

Participants in the class intervention included both girls and boys. While the potential beneficiaries of the intervention were young girls, boys were considered critical counterparts and germane to the cycle of intergenerational relationships as they get older.

The intervention was adapted from a trial in Kenya which showed a reduction in pregnancy of 28% [19] to the Botswana context over the course of six to 9 months. This process included multiple pilots, focus group discussions, and contextual adaptation in partnership with the Ministry of Education, the Ministry of Health, National AIDS Coordinating Agency, the University of Botswana, the Jameel Poverty Action Lab (J-PAL), Evidence Action, the largest HIV clinic in the country (Botswana-Baylor Children’s Clinical Center of Excellence), and a youth-led NGO (Young 1ove). J-PAL also conducted the randomization and Young 1ove and Baylor enrolled participants. The class was designed to be short and cost-effective to facilitate scale-up and integration into existing curricula. A Project Review Committee (PRC) chaired by the Ministry of Education and consisting of key stakeholders across the government, UN and civil society met before the intervention and periodically thereafter to advise the trial.

Fifteen youth facilitators were trained for 5 days to deliver the intervention. The facilitators were selected out of a competitive pool of young people with experience and the ability to address sensitive topics using a youth-friendly approach. Facilitator preparation included class and pilot training. One facilitator was allocated to each classroom during intervention, ranging from 31 to 40 students on average. Random monitoring visits were conducted during implementation to provide quality assurance.

### The control group

Students randomized to the control group received the standard-of-care for youth in schools, typically a life skills program delivered by the school’s Guidance and Counseling teacher. The curriculum covers sexual education and emphasizes abstinence messaging.

### Randomization

Schools were randomized using Stata 15 (STATACORP. College Station, TX). A random seed was set to enable replication of the randomization. The unit of randomization was the school. Randomization was stratified by region and school level (primary and secondary) to minimize the risk of contamination. All students in the designated grades (6, 8 and 9) were reached with either the intervention or a survey at baseline and a follow-up survey 12 months later. Study sensitizations were conducted with school heads to ensure community buy-in and minimize attrition. School heads were not blinded for logistical purposes and program planning. However, since schools are remote and far apart, the likelihood of cross-school contact is low. The average population density of Botswana is 3.5 people per square kilometer, the 11th lowest density country in the world.

### Data collection procedures

All procedures were approved by the Botswana Ministry of Health IRB, the Botswana Ministry of Education Research Department and the MIT IRB. All data analysts have a human subject certificate. Since both the intervention and survey were non-invasive in nature, the Ministry of Education provided a waiver to collect data with verbal informed consent from students and school administrators. Prior to data collection, surveyors made clear participation was voluntary and confidential. It was explained that consent was provided by voluntarily agreeing to fill in the relevant data forms. Parent consent was sought out and obtained where appropriate. The trial was registered with the Pan African Clinical Trials Registry (PACTR201901837047199) in December 2018. The trial registry did not occur in advance of participant enrollment due to ambiguity of eligibility given the applied nature of the study.

Surveys were paper-based and self-administered in each class. The survey was a short, anonymous, two-page questionnaire where students were asked to answer questions covering school information, student demographic information and questions about knowledge, attitudes and self-reported sexual behavior. Surveyors provided a short introduction, handed out the surveys, and were available to answer questions during the survey. The survey was written in English, the language of instruction in schools after grade 4. However, instructions were given in a combination of Setswana and English to ensure comprehension. Questions were also answered in Setswana when necessary.

In addition to a paper-based survey, a roll call was taken at baseline of all students who were present. These roll call registers were taken back to schools at follow-up and read out loud. Students were marked as missing if they were absent and classmates provided one of six rationales: pregnant, dropout, absent for two or more weeks, transfer, repeating a grade or unknown. This approach was used instead of the daily school roll calls conducted by school teachers since pilots revealed these rosters were completed inconsistently.

A survey was also administered to school administrators. At primary schools, this was conducted with the guidance & counseling teacher. At junior schools, this was conducted with one of the Heads of Departments (HOD). Guidance teachers and HODs were chosen as the administrators with the most accurate knowledge of missing girls. School heads are removed from day-to-day activity, and individual teachers are only knowledgeable about their individual classrooms. Guidance teachers and HODs are most likely to have a complete picture of the girls missing and why.

The school administrator survey asked the administrator to identify all missing girls and the rationale for their being missing along the same six sub-categories as the roll call: pregnancy, dropouts, absent for more than 2 weeks, transfer, repeated or unknown.

Administrator surveys were triangulated real-time with the roll call data. The lead surveyor at each school was instructed to check if both surveys aligned. If discrepancies existed, the administrator was asked to identify missing girls picked up by the roll call.

Administrative data was further collected from the Ministry of Education on baseline enrollment by gender as well as dropouts due to pregnancy. This data was used to compare characteristics across intervention and control groups and as a component of a vector of baseline controls in the analysis.

### Measures

Our primary outcome measure is pregnancy. The pregnancy measure was a maximum of pregnancies identified through the roll call and through the school administrator. This measure is an objective behavioral measure of risky sex, and is analogous to the measure used in a similar trial in Kenya enabling comparability.

Secondary outcomes were self-reported measures of sexual behavior at a 12-month follow up including: “Have you ever had sexual intercourse?” “How many people have you had sex with in the last 12 months?” and “Have you ever used a condom while having sex?” These measures were piloted in Botswana, build on a similar trial in Kenya, and relate to large-scale surveys conducted on sexual behavior such as the Demographic Health Survey and the Botswana AIDS Impact Survey.

### Analysis

Data were digitized from paper-based forms using Captricity software (Captricity. Oakland, CA). The data was manually checked. In only 1% of cases there were errors, such as text that was not legible by the software. These were manually corrected. Data was cleaned by a second reviewer. Intention to treat analysis was done using generalized linear multilevel models with a binomial distribution and log link at the individual level, clustering standard errors at the school level. Analyses compared the intervention and control groups at follow-up, adjusting for a vector of school-level baseline covariates to enhance statistical precision. Results are stratified by school level (primary and secondary) and by region. All statistical analysis was conducted in Stata 15 (STATACORP. College Station, TX). The analysis reported is conducted for girls, the primary beneficiary of the intervention. This provides an analogous comparison for self-reported measures of sexual behavior since the pregnancy outcome applies only to girls. The sample size fluctuates across variables given varying response rates.

## Results

### Individual baseline characteristics

Table 1 presents summary statistics by intervention group and school type for all students. The mean age of primary school students was 12.3 and 14.7 for junior secondary school students. The average class size in primary school was 31 and 40 in junior secondary school. Only 9–11% of students could identify 40-year old men as most likely to have HIV and only 42–45% of students could identify older partners as riskier in general. Faithfulness was the factor most salient in junior school in choosing a sexual partner at 50%, followed by condom use at 20%. In primary school, there was an even split between these two factors at around 30% of students choosing each. A partners’ HIV status was the top factor for roughly 14% of students. The percentage of students who had sex to date was 10–12%. Pregnancy rates at baseline were relatively low according to administrative data at .5% for junior school and nearly 0% at primary school.

**Table 1:** Baseline Characteristics by Intervention Group

	Junior Secondary School				Primary School			
	Intervention	Control	Overall	*n*	Intervention	Control	Overall	*n*
*Survey Data*								
Female	51.6%	50.6%	51.1%	17,287	50.2%	48.8%	49.5%	10,419
Age	14.7	14.7	14.7	16,297	12.4	12.1	12.3	9253
Class Size	40	40	40	17,412	30	31	31	10,575
School Size	439	426	432	17,412	88	88	88	10,575
School Absenteeism	5.2%	6.4%	5.8%	17,412	3.9%	3.3%	3.6%	10,575
Grade 6	–	–	–	–	90.4%	96.5%	93.6%	10,575
Grade 8	49.5%	51.2%	50.4%	17,412	–	–	–	–
Grade 9	48.6%	47.5%	48.1%	17,412	–	–	–	–
Decision Factor								
wealth	6.8%	7.7%	7.3%	17,028	10.4%	9.5%	9.9%	10,094
HIV status	14.0%	13.8%	13.9%	17,028	12.4%	15.4%	14.0%	10,094
social status	5.7%	5.9%	5.8%	17,028	7.5%	9.1%	8.4%	10,094
condom use	21.3%	19.4%	20.3%	17,028	34.8%	28.1%	31.2%	10,094
faithfulness	49.8%	50.2%	50.0%	17,028	31.5%	35.5%	33.6%	10,094
40 year-old men most likely to have HIV	9.1%	9.4%	9.3%	17,059	10.3%	10.7%	10.5%	10,153
Older partners are riskier	45.5%	44.3%	44.9%	17,062	42.7%	43.9%	43.3%	10,201
Self Efficacy	80.9%	81.7%	81.3%	17,412	63.0%	69.8%	66.6%	10,575
Ever had sex	12.2%	10.7%	11.4%	17,104	12.8%	10.3%	11.4%	10,248
Girls in class with older partners	1.39	0.81	1.08	15,916	1.21	0.36	0.73	9207
Classmates pregnant	0.12	0.15	0.13	15,976	0.07	0.07	0.07	9400
*Administrative Data from Ministry of Education*								
Female Enrollment	321	285	303	17,412	269	310	291	10,575
Male Enrollment	312	294	303	17,412	275	323	300	10,575
Total Enrollment	634	579	605	17,412	544	633	591	10,575
% pregnant	0.5%	0.5%	0.5%	17,412	0.0%	0.0%	0.0%	10,575

### Outcomes

Table 2 reports results at the 12-month follow up for girls. The intervention reduced pregnancy with an adjusted Relative Risk Ratio (aRRR) of .657 [95% CI .433–.997] significant the 5% level. Effects were larger at junior secondary school (aRRR = .575 [95% CI .394–.841]) and in rural areas (aRRR = .518 [95% CI .323–.831]) significant at the 1% level. There were no significant effects for primary school students. Self-reported outcomes reveal delay in sexual debut and fewer partners. Effects are largest in junior school. The biggest shift occurred in delaying sexual debut (aRRR = .786 [95% CI .641–.963]) significant at the 5% level. The second largest margin of sexual behavior change was fewer partners (aRRR = .793 [95% CI .643–.979]) significant at the 5% level. There is no significant reduction in an attempt to use a condom.

**Table 2:** Effect on Sexual Behavior - Comparing Intervention versus Control

	All	Rural	Junior	Primary
Pregnancy				
aRRR (95% CI)	0.657 (0.433 - 0.997)	0.518 (0.323 - 0.831)	0.575 (0.394 - 0.841)	0.557 (0.0539 - 5.748)
*p*-value	0.049	0.006	0.004	0.623
*n*	15,335	11,483	9902	5433
More than 1 Sexual Partner				
aRRR (95% CI)	0.953 (0.808 - 1.125)	0.916 (0.760 - 1.104)	0.793 (0.643 - 0.979)	0.953 (0.715 - 1.271)
*p*-value	0.568	0.358	0.031	0.745
*n*	14,130	10,595	9151	4979
Ever Had Sex				
aRRR (95% CI)	0.885 (0.757 - 1.034)	0.856 (0.714 - 1.028)	0.786 (0.641 - 0.963)	0.996 (0.716 - 1.386)
*p*-value	0.123	0.096	0.020	0.982
*n*	14,113	10,587	9131	4982
Ever Tried to Use a Condom				
aRRR (95% CI)	1.006 (0.958 - 1.057)	0.984 (0.935 - 1.037)	1.050 (0.990 - 1.114)	0.968 (0.893 - 1.049)
*p*-value	0.801	0.553	0.106	0.428
*n*	13,944	10,476	8993	4951

^a^Adjusted Relative Risk Ratios (aRRR) are calculated clustering standard errors by school and includes region and school level fixed effects to account for stratification. Adjustments were generated using a vector of school-level baseline control variables to enhance statistical precision. This vector includes: enrollment, dropout, class-reported meaures of pregnancy and class-reported rates of girls dating older partners. Due to limited pregnancy in primary schools, we include a specification without controls or region-fixed effects to enable inclusion of the full sample. The results are robust in effect size and significance to specification

Table 3 reports exploratory analysis of results by baseline beliefs on which partner students think is most likely to have HIV before the intervention. These are school-level averages split above or below the median. We see that in schools where students think 10–19-year-olds are lower risk to begin with, there is no effect (aRRR = .972 [95% CI .707–1.336]. For schools that think 10–19-year-olds are higher risk at baseline, there is a large and significant reduction in pregnancy (aRRR = .375 [95% CI .210–.669]. This reinforces the notion that the largest impact occurs for students who initially over-estimate the risk of 10–19-year-olds. This is likely since they learn new information and update their beliefs that similar-age partners are in fact a lower risk partner choice than older partners. We see a corresponding larger effect where students think the risk of older 20–29-year-old partners is low to begin with, since they are most likely to learn older partners are riskier.

**Table 3:** Effect on Pregnancy by Baseline Belief - Comparing Intervention versus Control

		% of students that think a given age group is high or low risk at baseline			
		10-19 year olds		20-29 year olds	
	All	Low Risk	High Risk	Low Risk	High Risk
Pregnancy					
aRRR (95% CI)	0.657 (0.433 - 0.997)	0.972 (0.707 - 1.336)	0.375 (0.210 - 0.669)	0.429 (0.194 - 0.951)	0.887 (0.568 - 1.384)
*p*-value	0.049	0.860	0.001	0.037	0.597
*n*	15,335	7118	8217	6880	6758

^a^Adjusted Relative Risk Ratios (aRRR) are calculated clustering standard errors by school and includes region and school level fixed effects to account for stratification. Adjustments were generated using a vector of school-level baseline control variables to enhance statistical precision. This vector includes: enrollment, dropout, class-reported meaures of pregnancy and class-reported rates of girls dating older partners

## Discussion

Results from this cluster-randomized controlled trial indicate that revealing the relative HIV risk of older partners and that same-age partners are a relatively safer sex option can change sexual behavior. This corroborates findings from a similar intervention in Kenya and suggests this risk-reduction approach could have significant effects on preventing risky sex, pregnancy and potentially new HIV infections. We observe heterogeneity in results, with larger effects in rural areas and in junior school. There are no significant effects in primary school. This is potentially driven by higher baseline rates of intergenerational sex and pregnancy in rural and junior school settings. In rural settings, this might also be since the likelihood of receiving other interventions is limited.

Moreover, we find sexual debut is delayed and girls report having fewer partners. However, self-reported condom use does not change. This may suggest a likely mechanism in reducing risky sex is either delayed sexual debut in general or greater partner selectivity and exclusivity with enhanced negotiating power to delay sexual debut.

We find that baseline beliefs affect the magnitude of effects substantially. The largest margin of impact is students who initially think 10–19 years are riskiest to begin with. This is potentially since these are the students most likely to learn they are in fact safer during the intervention, thereby getting both a ‘shock’ in general and introducing a novel safer sex alternative.

Taken together, this intervention is high-potential while heterogeneous along contextual factors, such as age of sexual debut and baseline beliefs of risk. Since the intervention is a 1-h module, it stands out for its cost-effectiveness. Back-of-the-envelope calculations suggest the intervention can avert a pregnancy for $480–$980 and an instance of unprotected sex for $24–$49.

Results of this study should be considered in light of several limitations. First, the measure of pregnancy used is not biological. Alternative measures of pregnancy were collected such as noting of visibly large stomachs but are excluded from the analysis since this measure is not validated in the literature. We were not able to conduct biological HIV tests due to cost and ethical concerns. While pregnancy is a behavioral outcome and a robust proxy for unprotected sex, it is an imperfect proxy for HIV. To this end, future research should collect biological outcomes on both pregnancy and HIV. A further limitation is the relatively short follow-up period. This limits our ability to ascertain the durability of the findings. A further limitation of this study is lack of reliable data on partner age to determine conclusively if girls shifted to dating same-age partners. While this question was included in surveys, response rates were too low to interpret.

We note that while our analysis centers on young girls, which were the focus of this study and comprise a majority of the HIV and pregnancy burden, young boys are also affected and are essential actors in successful intervention. We further note that results are heterogeneous. Adaptation and future testing should be conducted with this in mind. Moreover, the intervention targets a specific behavior change for a sub-set of girls who have agency over sexual partners. There are instances where sex is coerced. This intervention is unlikely to be effective for this sub-set of girls. We note that future studies should consider the potential for adverse events and measure outcomes accordingly. This intervention does not address the totality of issues young people face. It should be seen as a cost-effective complement to comprehensive sexuality education.

## Conclusion

This study contributes to the limited evidence base on risk reduction approaches in sexual education in sub-Saharan Africa. We provide rigorous evidence that revealing young people are a safer sex alternative to riskier older partners is a promising and cost-effective approach. The intervention has heterogeneous effects and should be adapted, contextualized and tested with this in mind.

## Abbreviations

aRRR: Adjusted Relative Risk Ratio
ARV: Antiretrovirals
HIV: Human Immunodeficiency Virus

## References

[1] Joint United Nations Programme on HIV/AIDS (UNAIDS) (2018). Global HIV & AIDS statistics – 2018 fact sheet.

[2] Joint United Nations Programme on HIV/AIDS (UNAIDS) (2017). UNAIDS Data 2017 Reference Report.

[3] Joint United Nations Programme on HIV/AIDS (UNAIDS) (2014). The gap report.

[4] UNESCO. International technical guidance on sexuality education: an evidence-informed approach for schools, teachers and health educators. Paris: UNESCO; 2009.

[5] Fiore MC, Smith SS, Jorenby DE, Baker TB (1994). The effectiveness of the nicotine patch for smoking cessation: a meta-analysis. JAMA..

[6] Potier C, Laprévote V, Dubois-Arber F, Cottencin O, Rolland B (2014). Supervised injection services: what has been demonstrated? A systematic literature review. Drug Alcohol Depend.

[7] Ng J, Sutherland C, Kolber MR (2017). Does evidence support supervised injection sites?. Can Fam Physician.

[8] Underhill K, Montgomery P, Operario D (2007). Sexual abstinence only programmes to prevent HIV infection in high income countries: systematic review. BMJ..

[9] Aral SO, Peterman TA (1996). Measuring outcomes of behavioural interventions for STD/HIV prevention. Int J STD AIDS.

[10] Kamali A, Kinsman J, Nalweyiso N, Mitchell K, Kanyesigye E, Kengeya-Kayondo JF, Carpenter LM, Nunn A, Whitworth JA (2002). A community randomized controlled trial to investigate impact of improved STD management and behavioural interventions on HIV incidence in rural Masaka, Uganda: trial design, methods and baseline findings. Tropical Med Int Health.

[11] Gallant M, Maticka-Tyndale E (2004). School-based HIV prevention programmes for African youth. Soc Sci Med.

[12] Paul-Ebhohimhen VA, Poobalan A, Van Teijlingen ER (2008). A systematic review of school-based sexual health interventions to prevent STI/HIV in sub-Saharan Africa. BMC Public Health.

[13] Kirby DB, Laris BA, Rolleri LA (2007). Sex and HIV education programs: their impact on sexual behaviors of young people throughout the world. J Adolesc Health.

[14] Doyle AM, Ross DA, Maganja K, Baisley K, Masesa C, Andreasen A, Plummer ML, Obasi AI, Weiss HA, Kapiga S, Watson-Jones D (2010). Long-term biological and behavioural impact of an adolescent sexual health intervention in Tanzania: follow-up survey of the community-based MEMA kwa Vijana trial. PLoS Med.

[15] Mavedzenge SM, Doyle AM, Ross DA (2011). HIV prevention in young people in sub-Saharan Africa: a systematic review. J Adolesc Health.

[16] Bandiera O, Buehren N, Burgess R, Goldstein M, Gulesci S, Rasul I, Sulaiman M. Women’s empowerment in action: evidence from a randomized control trial in Africa. Washington D.C.: World Bank; 2017.

[17] Ross DA, Dick B, Ferguson J, World Health Organization. Preventing HIV/AIDS in young people: a systematic review of the evidence from developing countries. Geneva: World Health Organization 16921915

[18] Lo NC, Lowe A, Bendavid E (2016). Abstinence funding was not associated with reductions in HIV risk behavior in sub-Saharan Africa. Health Aff.

[19] Dupas P (2011). Do teenagers respond to HIV risk information? Evidence from a field experiment in Kenya. Am Econ J Appl Econ.

[20] Statistics Botswana. Preliminary results: Botswana AIDS impact survey IV (BAIS IV), 2013. Gaborone: Statistics Botswana; 2013.

[21] Botswana Ministry of Basic Education. Second Botswana Youth Risk Behavioural and Biological Surveillance Survey Report (2016).

[22] Nkosana J, Rosenthal D (2007). The dynamics of intergenerational sexual relationships: the experience of schoolgirls in Botswana. Sex Health.

[23] Olson R (2012). Refocusing the Lens: recognizing and enhancing agency around HIV risk avoidance with adolescent girls in eastern and southern Africa.

[24] United Nations (2017). Department of Economic and Social Affairs, Population Division. World Population Prospects: The 2017 Revision, Key Findings and Advance Tables. Working Paper No. ESA/P/WP/248.

[25] Joint United Nations Programme on HIV/AIDS (UNAIDS) (2013). Global Report: UNAIDS report on the global AIDS epidemic 2013.

